# Puerarin attenuates diabetic kidney injury through the suppression of NOX4 expression in podocytes

**DOI:** 10.1038/s41598-017-14906-8

**Published:** 2017-11-03

**Authors:** Xueling Li, Weijing Cai, Kyung Lee, Bohan Liu, Yueyi Deng, Yiping Chen, Xianwen Zhang, John Cijiang He, Yifei Zhong

**Affiliations:** 1 Division of Nephrology, Longhua Hospital, Shanghai University of Traditional Chinese Medicine, Shanghai, China; 2 Department of Medicine, Division of Nephrology, Icahn School of Medicine at Mount Sinai, NY, USA; 3 Renal Section, James J Peters VAMC, Bronx, NY USA

**Keywords:** Chronic kidney disease, Diabetic nephropathy

## Abstract

*Radix puerariae*, a traditional Chinese herbal medication, has been used to treat patients with diabetic nephropathy (DN). Several studies demonstrated that puerarin, the active compound of *radix puerariae*, reduces diabetic injury in streptozotocin (STZ)-induced diabetic rodent models. However, as STZ injection alone results in mild kidney injury, the therapeutic benefit afforded by puerarin in DN remained inconclusive. Thus we sought to clarify the role of puerarin by employing an accelerated DN model, STZ-induced diabetes in the endothelial nitric oxide synthase-null (eNOS^−/−^) mice. Puerarin treatment of diabetic eNOS^−/−^ mice significantly attenuated albuminuria and diabetic kidney injury, which were associated with reduced oxidative stress and reduced NAPDH oxidase 4 (NOX4) in glomeruli of diabetic eNOS^−/−^ mice. Puerarin treatment of murine podocytes culture in high glucose conditions led to reduced superoxide production and NOX4 expression. We further determined that that puerarin treatment increased both mRNA and protein levels of SIRT1 in podocytes and that puerarin led to SIRT1-mediated deacetylation of NF-κB and suppression of NOX4 expression. Our findings confirm the renoprotective effects of puerarin in an experimental model of advanced DN and provide a molecular mechanism by which puerarin exerts the anti-oxidative effects in podocytes  in the diabetic milieu.

## Introduction

Diabetic nephropathy (DN) remains the most common cause of end-stage renal disease (ESRD) in US and worldwide^1,2^. The incidence of DN has also dramatically increased in China in the recent years due to the increasing prevalence of diabetes, and DN may soon become the leading cause of ESRD in China^3,4^. The treatment of DN has been limited to hyperglycemic control, blood pressure control, and renin-angiotensin system blockade. However, many patients on angiotensin converting enzyme inhibitors (ACEI) or angiotensin receptor blockades (ARB) continue to progress to ESRD^5^. Moreover, new clinical trials in patients with DN have either failed to show efficacy or were prematurely terminated due to significant side effects^6,7^, such that there has not been a new FDA-approved therapy against DKD progression for the past 20 years. Therefore, there is a large unmet need to develop potent and safer therapies against DN.

The early stage of DN is clinically defined by the appearance of persistent microalbuminuria^8,9^. Podocyte injury is a major contributor to the development of microalbuminuria in DN^10^, and the reduction in podocyte density is the strongest predictor of progressive DN^11,12^. Apoptosis and detachment from the glomerular basement membrane have been proposed as potential mechanisms of podocyte loss^13^, and oxidative stress is considered to be a major culprit in podocyte injury in DN^14^. Among the different NADPH oxidase (NOX) isoforms, NOX4 was shown to be the main enzyme contributing to the increased oxidative stress in podocytes, as either the genetic ablation of *Nox4* or pharmacological inhibition of its activity was shown to attenuate DN in a rodent model of diabetes^15^. Further, podocyte-specific deletion of NOX4 was also shown to mitigate diabetes-induced podocyte injury and to confer renoprotection in DN^16^.

Traditional Chinese herbal medications have been used widely in China to treat patients with DN. However, their mechanisms of renoprotection in DN remain unclear. We previously reported that Chen’s Tangshen decoction, in which puerarin is a major component, is able to significantly reduce microalbuminuria in patients with early DN^17^. A recent meta-analysis suggests that treatment of DN patients with puerarin induces a further reduction of albuminuria in combination with ACEI^18^. We and others have also shown that puerarin improve albuminuria in rodent models of streptozotocin (STZ)-induced diabetes^19–21^, although the extent of kidney injury was relatively mild in these models to clearly delineate the renoprotective effects of puerarin. Several studies have also shown both *in vitro* and *in vivo* that puerarin exhibited an anti-oxidative activity^19,20,22,23^, and that it attenuated apoptosis of proximal tubular cells through the restoration of mitochondrial function^24,25^. However the detailed mechanisms by which puerarin exerts renoprotection in DN had not been explored. Here, we sought to confirm the renoprotective effects of puerarin in an accelerated DN mouse model and to explore the mechanism by which puerarin attenuates oxidative stress and podocyte injury under diabetic conditions.

## Results

### Puerarin treatment attenuates proteinuria and kidney injury in the diabetic eNOS^−/−^ mice

Diabetes was induced in 6-week old eNOS^−/−^ mice using low-dose streptozotocin as described previously^26^, and age-matched citrate buffer-injected eNOS^−/−^ mice served as controls. Both diabetic and control mice were treated with either puerarin (20 mg/kg of body weight) or vehicle starting at 10 weeks post- STZ injection when a significant albuminuria is present in diabetic mice. All mice were sacrificed at the age of 18 weeks for assessment of renal function and histopathology. As shown in the Table 1, there were no significant differences in blood glucose between mice treated with puerarin or vehicle in either non-diabetic controls or in diabetic mice. The kidney-to-body weight ratio increased in diabetic eNOS^−/−^ mice in comparison to control eNOS^−/−^ mice, but this increase was significantly attenuated in puerarin-treated diabetic eNOS^−/−^ mice (Table 2).

**Table 1:** Blood glucose at baseline and during puerarin/vehicle treatment.

	Baseline		Treatment				
	Pre- injection	Post- injection	WK0	WK1	WK2	WK3	WK4
control eNOS^−/−^ +Vehicle	91 ± 5	88 ± 6	93 ± 7	95 ± 3	95 ± 8	96 ± 7	98 ± 9
control eNOS^−/−^ +Puerarin	90 ± 7	93 ± 8	93 ± 8	95 ± 8	92 ± 4	98 ± 9	99 ± 12
diabetic eNOS^−/−^ +Vehicle	96 ± 10	489 ± 48***	482 ± 56***	456 ± 32***	388 ± 79***	399 ± 62***	422 ± 15***
diabetic eNOS^−/−^ +Puerarin	110 ± 9	456 ± 39***	455 ± 67***	444 ± 22***	389 ± 73***	370 ± 38***	432 ± 48***

***P < 0.001 when compared to control eNOS^−/−^ mice (n = 6 in each group).

**Table 2:** Kidney-to-body weight ratio of mice at 18 weeks post-STZ/vehicle injection.

	control eNOS^−/−^ +Vehicle	control eNOS^−/−^ +Puerarin	diabetic eNOS^−/−^ +Vehicle	diabetic eNOS^−/−^ +Puerarin
Kidney/BW (mg/g)	8.08 ± 0.52	7.88 ± 0.48	13.24 ± 0.78***	9.12 ± 0.88*^,###^

*P < 0.05 and ***P < 0.001 when compared to control eNOS^−/−^ mice; ^###^P < 0.001 when compared to vehicle-treated diabetic eNOS^−/−^ mice (n = 6 in each group).

**Figure 1:**
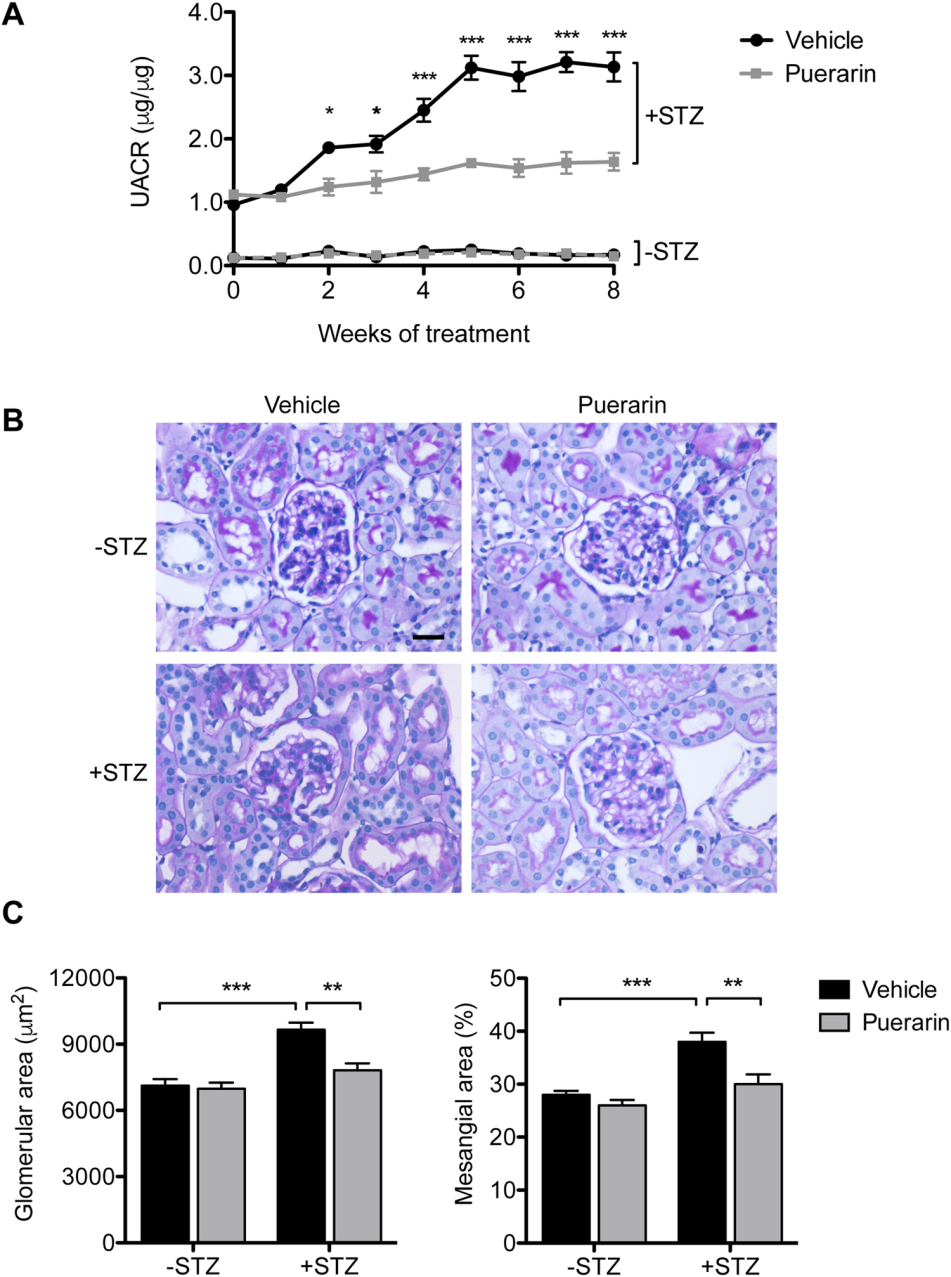
Puerarin reduced albuminuria and kidney injury in STZ-induced diabetic eNOS knockout mice. (**A**) Both diabetic and non-diabetic mice were treated with either vehicle or puerarin when mice developed albuminuria for a total of 9 weeks. Urine samples were collected weekly after starting the treatments for determination of urinary albumin/creatinine ratio. Significant differences were observed starting at 2 weeks after puerarin treatment compared with vehicle-treated diabetic mice. *p < 0.05 and *p < 0.001 compared to vehicle-treated diabetic mice, n = 6. (**B**) Representative image of periodic acid-Schiff (PAS) stained kidneys. Scale bar: 20 μm. (**C**,**D**) Morphometric analysis was performed to determine the glomerular area (**C**) and % of mesangial area (**D**) in the glomerular cross sections. **p < 0.01 and ***p < 0.001, n = 6.

As expected, urinary albumin to creatinine ratio (UACR) increased nearly 10-fold in diabetic eNOS^−/−^ mice compared to control eNOS^−/−^ mice (Fig. 1A). However, administration of puerarin significantly reduced the UACR and halted the further worsening of albuminuria over time in diabetic eNOS^−/−^ mice (Fig. 1A). Histologically, we also observed a significant attenuation of glomerular hypertrophy and mesangial expansion in puerarin-treated diabetic mice in comparison to vehicle-treated diabetic mice (Fig. 1B,C), indicating that puerarin treatment reduced the glomerular injury in diabetic eNOS^−/−^ mice.

### Puerarin reduces oxidative stress in diabetic mice

**Figure 2:**
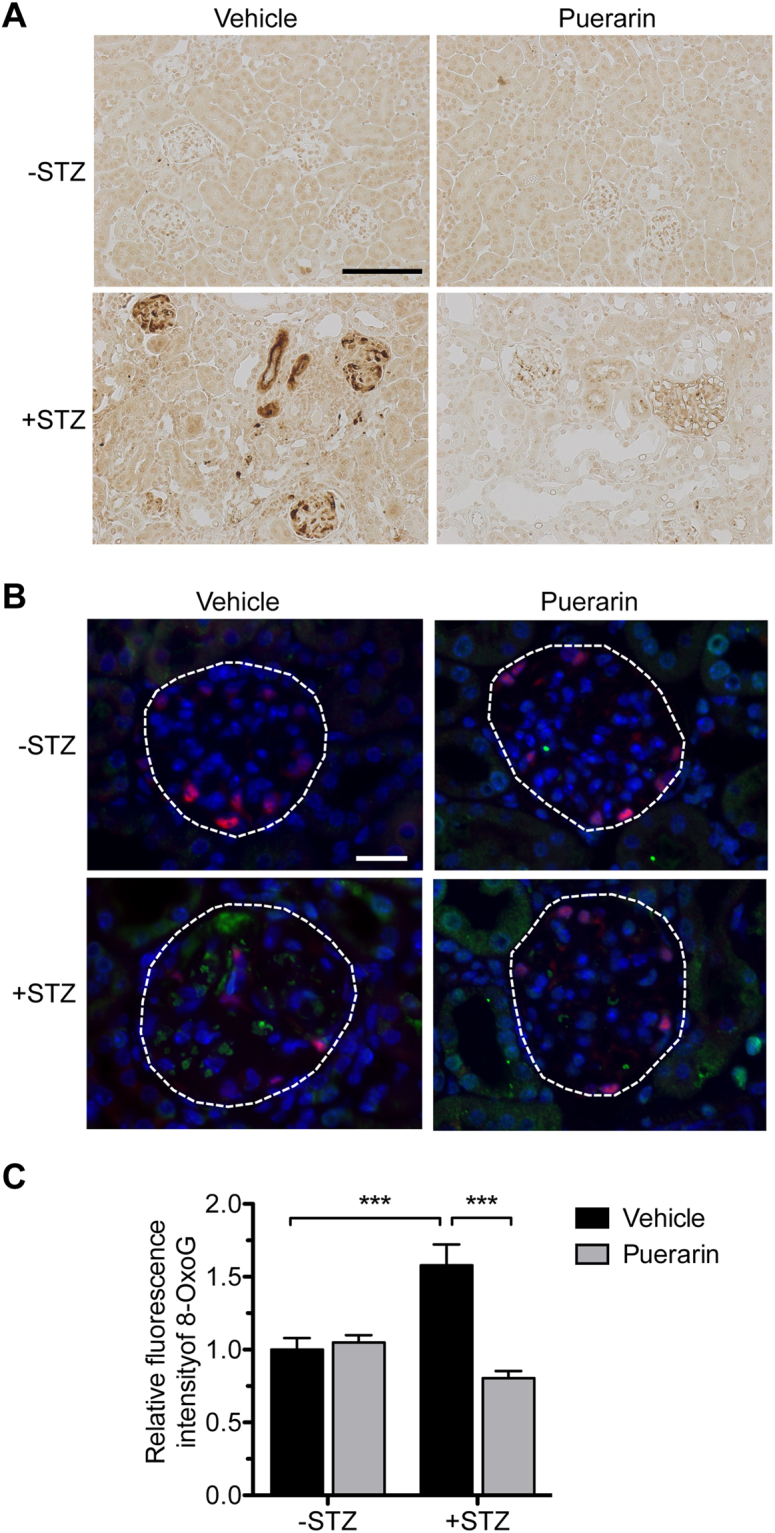
Puerarin improved oxidative stress in diabetic glomeruli. (**A**) Representative image of nitro-tyrosine immunostaining in paraffin-embedded kidney sections. Scale bar: 100 μm. (**B**) Representative images of 8-oxoG (green) and podocyte marker WT1 (red) immunofluorescence. DAPI was used as a counterstain. Scale bar: 20 μm. (**C**) Quantification of 8-oxoG immunofluorescence are shown. ***p < 0.001, n = 6 mice.

We next determined the effects of puerarin on the extent of oxidative stress in the diabetic glomeruli. Immunohistochemical staining showed a strong upregulation of nitrotyrosine expression in the glomeruli of vehicle-treated diabetic mice, which was not observed in nondiabetic control kidneys (Fig. 2A). Puerarin treatment led to a marked decrease of nitrotyrosine staining in the diabetic mouse kidneys (Fig. 2A). The attenuation of oxidative stress by puerarin was further confirmed by immunofluorescence staining of 8-oxoguanine (8-oxoG), a common DNA lesion resulting from oxidative stress, with co-staining of WT-1, a podocyte marker (Fig. 2B). A large extent of 8-oxoG-positive cells was also WT-1-positive, confirming an increase of oxidative stress in podocytes in diabetic milieu. We did note however that 8-oxoG-positive cells were not limited to podocytes, but were present in other glomerular cells in the diabetic eNOS^−/−^ kidneys. Puerarin treatment significantly decreased the number of 8-oxoG-positive cells, suggesting that puerarin improves DN through the inhibition of oxidative stress. Figure 2C shows the quantification of the 8-oxoG and WT-1 staining in the nondiabetic and diabetic kidneys.

**Figure 3:**
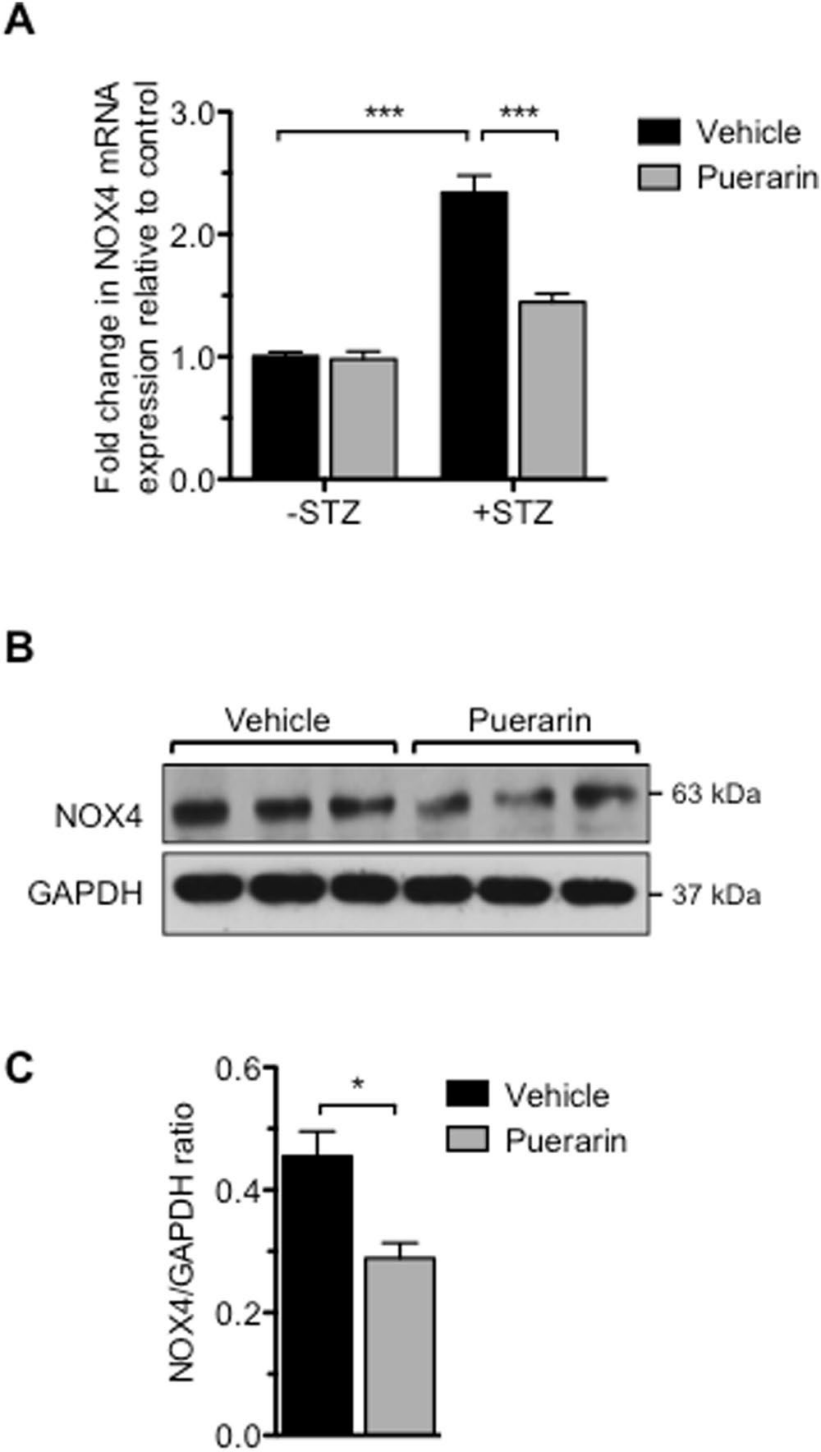
Treatment of puerarin reduced expression of NOX4 in the glomeruli of diabetic mice. (**A**) Real-time PCR analysis of NOX4 mRNA levels from isolated glomeruli. **p < 0.01 and ***p < 0.001, n = 6 mice. (**B**) Representative western blot analyses showing NOX4 expression in glomerular lysates. (**C**) Quantification of NOX4 protein expression normalized to GAPDH. *p < 0.05, n = 6 mice.

Because NOX4 is a main contributor of reactive oxygen species in the diabetic kidneys^15,16^, we next determined the mRNA and protein levels of NOX4 in the glomeruli of diabetic and nondiabetic mice treated with puerarin or vehicle. We found that NOX4 expression was indeed increased in vehicle-treated diabetic mice, but suppressed in puerarin-treated diabetic mice (Fig. 3A–C), indicating that puerarin attenuates oxidative stress through the inhibition of NOX4 expression.

### Puerarin reduces oxidative stress in conditionally immortalized murine podocytes

**Figure 4:**
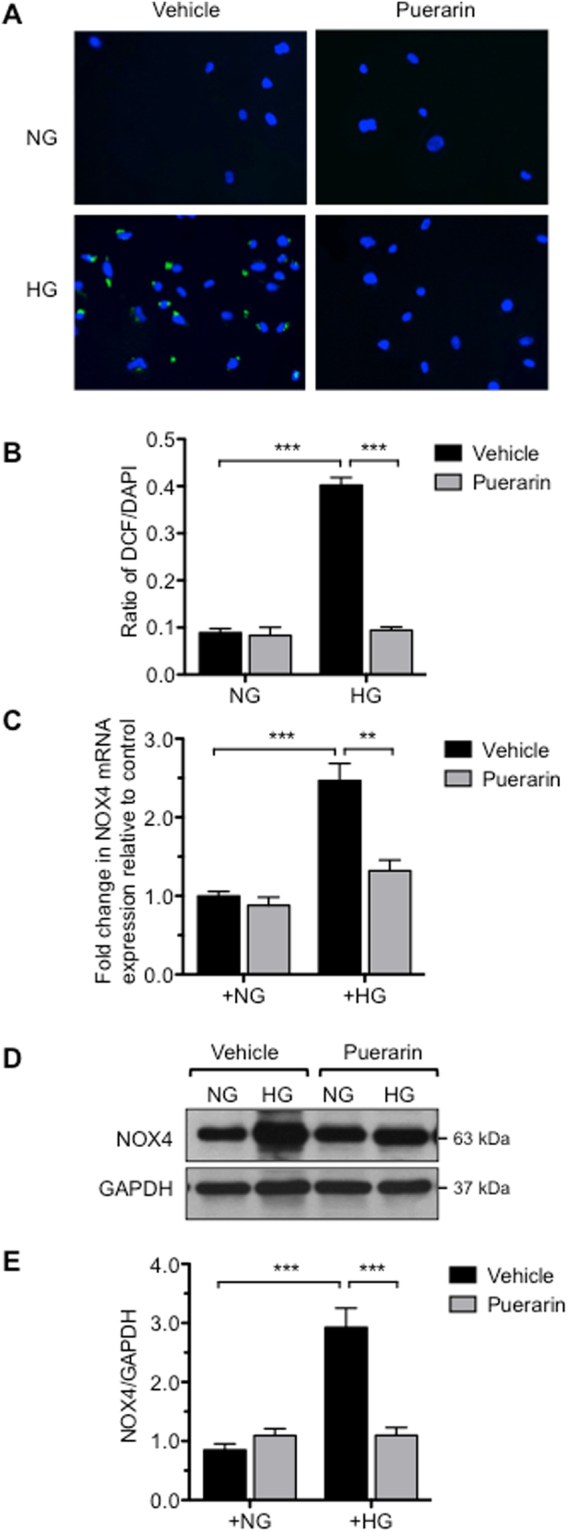
Puerarin inhibited oxidative stress in murine podocytes cultured in high glucose. (**A,B**) Immortalized mouse podocytes were cultured in either normal glucose (NG: 5 mM glucose + 25 mM mannitol) or high glucose (HG: 30 mM glucose) treated with DMSO vehicle or 10 μM puerarin for 24 hours. Superoxide production was determined by incubation of cells with dichlorofluorescin diacetate (DCFDA) fluorogenic dye and by detection of oxidized 2′, 7′-dichlorofluorescein (DCF). Puerarin reduced superoxide generation in podocytes cultured in high glucose media. Representative image is shown in (**A**) and ratio of DCF+ cells/total (DAPI+) cells per field are shown in (**B**) (***p < 0.001, n = 3, 7 fields per group). (**C**) Real-time PCR analysis of NOX4 mRNA levels in podocytes cultured with NG or HG treated with vehicle or 10 μM puerarin for 4 hours. GAPDH mRNA was used as an internal control. ***p < 0.001, n = 3. (**D**) Representative western blot analyses of NOX4 expression in podocytes cultured in NG or HG conditions for 24 hours. The representative blots of three individual experiments are shown here. (**E**) Quantification of NOX4 protein expression normalized to GAPDH is shown. **p < 0.01 and ***p < 0.001, n = 3.

In order to confirm the above *in vivo* findings, we tested *in vitro* whether puerarin can directly reduce the superoxide production in podocytes cultured under the high glucose condition. Reactive oxygen species were detected using the cell permeant 2′, 7′ –dichlorofluorescin diacetate (DCFDA), a fluorogenic dye. Detection of oxidized 2′, 7′ –dichlorofluorescein (DCF) showed that puerarin treatment reduced the superoxide production in podocytes treated with high glucose (Fig. 4A,B). Furthermore, we found that puerarin suppressed NOX4 expression at both mRNA and protein levels (Fig. 4C–E). Together, these data suggest that puerarin inhibits NOX-dependent superoxide generation in podocytes cultured in high glucose condition.

### Puerarin regulates NOX4 expression through SIRT1-NF-κB pathway in podocytes

**Figure 5:**
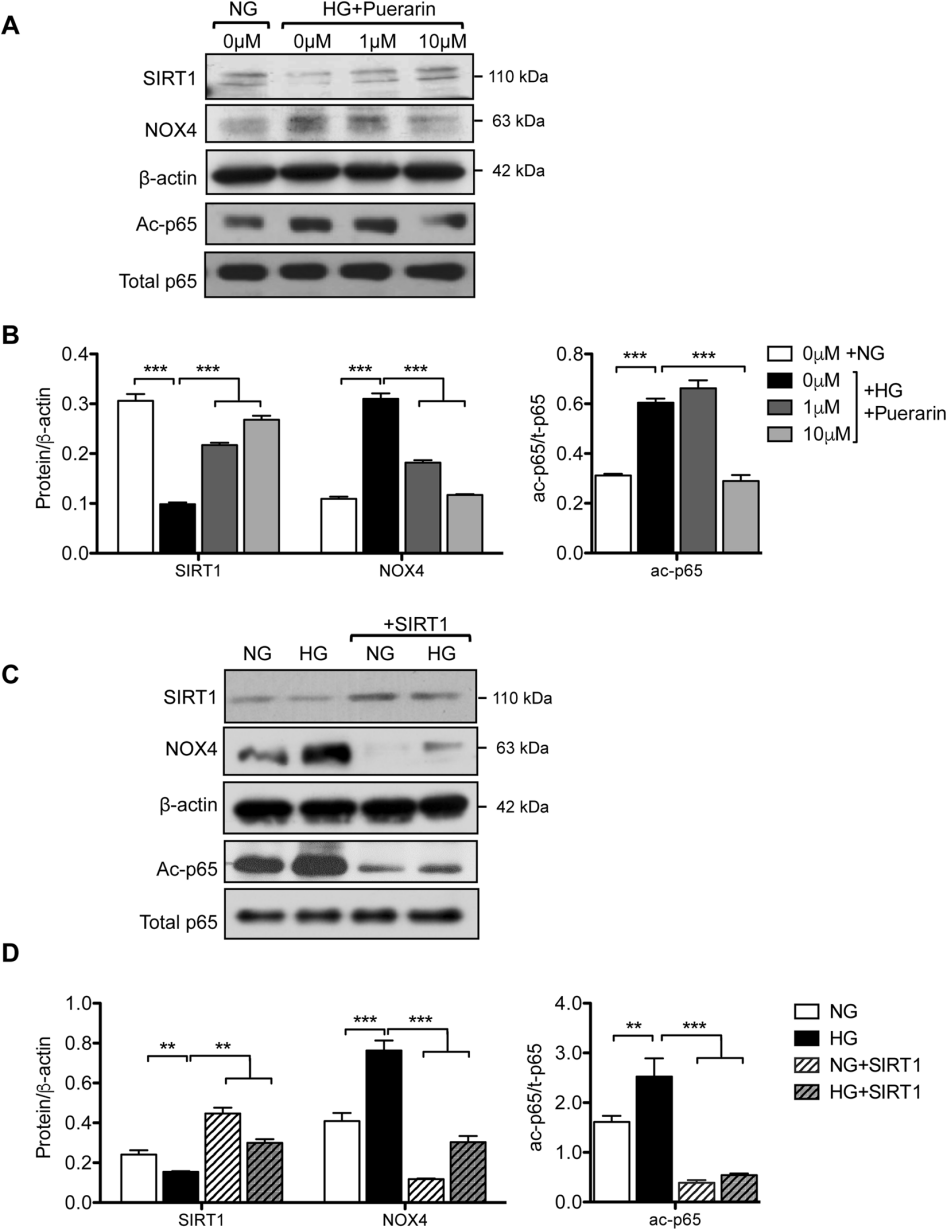
Puerarin downregulated NOX4 expression through activation of SIRT1 in murine podocytes exposed to high glucose. (**A,B**) Murine podocytes cultured in NG or HG were treated with different amounts of puerarin as indicated. The cells were lysed for western blot analysis for SIRT1, NOX4, acetyl-p65, total-p65, and β-actin. Representative blots of three independent experiments are shown in A, and quantification is shown in (**B**). (**C**,**D**) Podocytes transfected with SIRT1 overexpression vector or control vector were cultured in NG and HG for 24 hours. The cells were lysed for western blot analysis for SIRT1, NOX4, acetyl-p65, total-p65, and β-actin. Representative blots of three independent experiments are shown in (**C**), and quantification is shown in (**D**).

**Figure 6:**
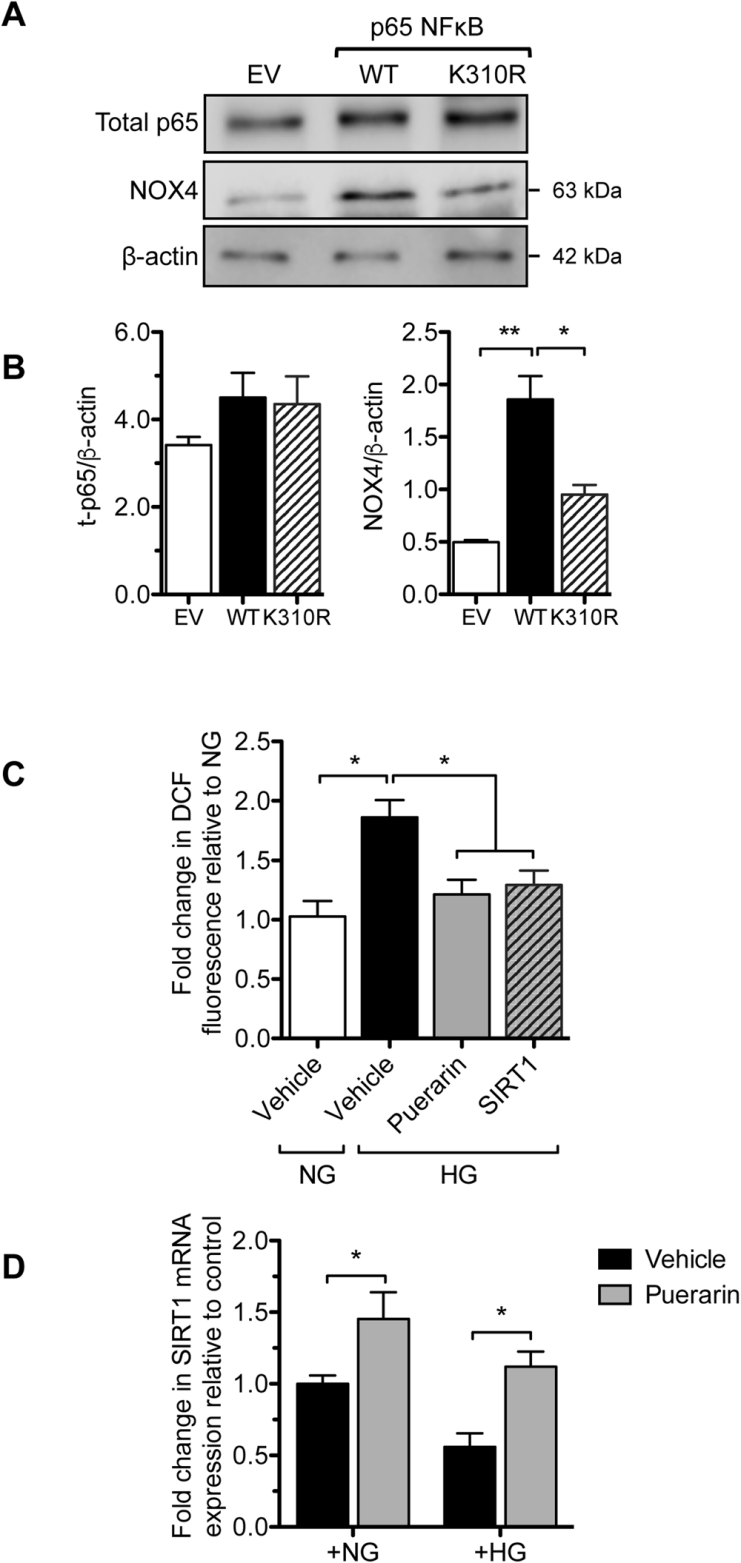
Puerarin downregulated NOX4 expression through deacetylation of p65NF-κB in murine podocytes exposed to high glucose. (**A,B**) Podocytes transfected with either wildtype p65 (WT), p65 with mutation of K310 acetyl-residue (K310R) or control empty vector (EV) were treated with TNF-α for 24 hours. Cells were lysed for western blot analysis for p65, NOX4 and β-actin. Representative blots of three independent experiments are shown in A, and quantification is shown in (**B)**. (**C**) Podocytes cultured in NG or HG were treated with 10 μM puerarin or 1 μM SIRT1 agonist SRT1720 for 24 hours. Superoxide production was then measured by the DCF fluorescence method. *p < 0.05 compared between groups n = 3. (**D**) Real-time PCR analysis of Sirt1 mRNA in podocytes treated with vehicle or puerarin in NG or HG conditions show increased Sirt1 expression by puerarin. *p < 0.05 compared between groups n = 3.

We previously found that the expression of NAD^+^-dependent histone deacetylase SIRT1 is reduced in human DN glomeruli^27^. Interestingly, we observed that the high-glucose-mediated suppression of SIRT1 protein expression in podocytes was alleviated by puerarin treatment at both low and high doses (1 and 10 μM, respectively) (Fig. 5A,B). Moreover, SIRT1 expression was inversely correlated with NOX4 expression (Fig. 5A,B), suggesting that SIRT1 negatively regulates NOX4 expression. Our earlier work showed that the activity of NF-κB is increased in podocytes in DN and that SIRT1 inhibits NF-κB activity through deacetylation^28^. We also observed that puerarin treatment led to the decreased NF-κB acetylation, likely through the stimulation of SIRT1 expression (Fig. 5A,B). In support of this, overexpression of SIRT1 in podocytes attenuated both NF-κB acetylation and NOX4 expression (Fig. 5C,D). Promoter analysis of NOX4 confirmed the presence of putative NF-κB binding sites, and others have shown in non-renal cells that NF-κB directly binds to the NOX4 promoter and regulates NOX4 expression^29^. Our previous studies indicated that NF-κB mutant with a lysine to arginine mutation of its acetylation site at Lys310 (p65^K310R^) serves as a dominant negative to block the activity of endogenous NF-κB^28^. We found that the overexpression of p65^K310R^ mutant (K310R) in podocytes led to the reduced NOX4 expression as compared to overexpression of wildtype p65 (WT) (Fig. 6A,B), strongly suggesting that NF-κB acetylation is required for NOX4 expression and that SIRT1 suppresses NOX4 expression through deacetylation of NF-κB. We further confirmed that the overexpression of SIRT1 exhibited similar inhibition of superoxide production in podocytes as puerarin (Fig. 6C). Lastly, we found that puerarin also increased SIRT1 mRNA levels in podocytes, suggesting that puerarin stimulates SIRT1 expression at the transcriptional level (Fig. 6D).

## Discussion

In the current study, we confirmed that puerarin mitigates albuminuria and kidney injury in STZ-induced diabetic mice with eNOS-deficiency, a model which better mimics the advanced human DN. In addition, we showed that the anti-oxidative effect of puerarin is mediated through the suppression of NOX4 expression in diabetic kidney *in vivo* and in cultured murine podocytes *in vitro*. We also demonstrated that puerarin suppressed NOX4 expression through the upregulation of SIRT1, resulting in increased deacetylation of NF-κB, thus uncovering a new molecular mechanism by which puerarin exerts renoprotection in kidney cells under diabetic conditions. Lastly, we have shown that puerarin stimulates SIRT1 expression at the transcriptional level.

Puerarin is a major isoflavonoid component from the root of *pueraria candollei* of *Leguminosae* family. Puerarin has a structure of 7-hydroxy-3-(4-hydroxyphenyl)-1-benzopyran-4-one-8-β-D-glucopyranoside^30^. Although large randomized clinical trials are lacking, several small clinical studies suggest that puerarin treatment significantly reduced albuminuria in patients with the stage 3 DN^18^. The renoprotective effects of puerarin have been also reported in several animal studies^19–21^. However, these studies were reported in STZ-induced diabetic rodent models with very mild kidney injury. In this study, we took advantage of the accelerated DN in absence of eNOS in STZ-induced diabetic eNOS^−/−^ mice^31^, which is a better-suited DN model to mimic the human diabetic kidney disease, to assess the effects of puerarin in DN progression. Using this advanced DN model, our results indeed confirm that the puerarin treatment commencing after the establishment of DN by detection of albuminuria significantly halted the progression of DN in diabetic eNOS^−/−^ mice. These results are consistent with previous clinical findings^18^, and strongly suggest the potential therapeutic benefits of puerarin treatments in DN patients.

Anti-oxidative activity of puerarin has been demonstrated in cardiovascular^32^ and neurological diseases^33^. Several studies also suggest that puerarin has anti-oxidative effects in kidney cells^19,20,22,23^. However, the mechanism by which puerarin exerts these effects remained unclear. Here, we report that the anti-oxidative effect of puerarin to confer renoprotection is in part mediated through the suppression of NOX4 expression in podocytes. Previous studies have shown that NOX4 expression is increased in diabetic kidneys and that podocyte-specific knockout of NOX4 attenuates DN^15,16^, underscoring the importance of NOX4 regulation in DN. In addition to NOX4 suppression, our results demonstrated that puerarin upregulates SIRT1 expression. The role of SIRT1 in DN has been well documented^28,34,35^. We have previously demonstrated the anti-inflammatory role of SIRT1 via deacetylation of NF-κB in DN^28^. Our data now suggest that puerarin decreases NOX4 expression through the inhibition of NF-κB activity by SIRT1 upregulation. Our promoter analysis further confirmed that NOX4 has binding sites for NF-κB. Consistent with our data, previous studies suggest that NF-κB directly mediates NOX4 expression^36,37^.

In line with these findings, recent studies reported that SIRT1 expression is increased in the diabetic kidney from mice treated with puerarin^20^. However, it remained unclear how puerarin regulates SIRT1 in the kidney cells. Here, we found that puerarin can upregulate both mRNA and protein levels of SIRT1 in podocytes, suggesting a transcriptional regulation of SIRT1. Since SIRT1 regulates multiple biological pathways such as aging,^38^, metabolism^39^, cancer and inflammation^40^, we believe that puerarin and its analogues could have a broader application in its therapeutic usage. Moreover, the renoprotective effects of puerarin may not be limited to anti-oxidative effects. It has been shown that puerarin can attenuate the apoptosis of proximal tubular cells through the restoration of mitochondrial function^25,41^. We have previously shown that puerarin improves DN through regulation of metalloproteinase 9 (MMP9) in podocytes^19^.

In summary, we confirmed the renoprotective effects of puerarin treatment in a mouse model with established DN. We demonstrated that puerarin exerts anti-oxidative effects through the activation of SIRT1-mediated NF-κB deacetylation and that puerarin is a stimulator of SIRT1 expression in podocytes. Our study suggests that puerarin may be a potential drug to treat patients with DN. However, future clinical studies are required to confirm the therapeutic effects of puerarin in patients with DN.

## Methods

### Animal studies

8-week old eNOS-null male mice on a C57BL/6 background were purchased from The Jackson Laboratory (Bar Habor, ME). Diabetes was induced by intraperitoneal injection of freshly prepared streptozotocin (STZ) (Sigma-Aldrich, Saint Louis, MO) dissolved in 0.1 M citrate buffer, pH 4.5 at 50 mg/kg after 4–6 hours of food withdrawal for 5 consecutive days. Control mice were injected with sodium citrate buffer. Blood glucose was measured every week. 2 weeks after diabetes was confirmed in mice (blood glucose > 250 mg/dl), mice were given puerarin (Sigma-Aldrich, Saint Louis MO) dissolved in 5% DMSO by oral gavage at a dose of 20 mg/kg body weight/day, or 5% DMSO vehicle as control, for 8 weeks. Urine samples were collected every week until they were sacrificed. All experimental methods were performed in accordance with the approved guidelines as described in the Guide for the Care and Use of Laboratory Animals^42^. All animal studies were approved by the Institutional Animal Care and Use Committee at the Icahn School of Medicine at Mount Sinai, New York, NY.

### Urine albumin and creatinine measurement

Urine albumin was measured using an ELISA kit (Bethyl Laboratory, Houston, TX), and urine creatinine was measured using a colorimetric assay kit (Cayman, Ann Arbor, MI).

### Kidney histology

Kidney samples were fixed in 10% formalin at room temperature for 16 hours and embedded in paraffin. Tissues were cut into 4 μm thick sections for periodic acid–Schiff (PAS) staining. Glomerular area and percentage of glomerular area were calculated as described (2).

### Immunohistochemistry and immunofluorescence staining

Paraffin-embedded kidney sections were deparaffinized and rehydrated for immunostaining. Specific antibodies used in this study are as following: anti-nitro-tyrosine from Santa Cruz (SC-32757) at 1:100 dilution, anti-8-oxo-G from Japan Institute for the Control of Aging (N45.1) at 1:50 dilution, and anti-WT-1 antibody was from Santa Cruz at 1:50 dilution. The secondary and tertiary antibodies were obtained from Jackson Immunoresearch, Inc. and used at 1:200 dilution.

### Glomerular RNA isolation

Mice glomeruli were isolated as previously reported (1). Total RNA was extracted from glomeruli using Trizol method. Purified RNA underwent DNA digestion using RNase-free DNase set (79254, Qiagen, Germantown, MD).

### Cell culture

Conditionally immortalized murine podocytes were obtained from Dr. Peter Mundel and cultured at 37 °C for 7 days for full differentiation before being used as described^43^.

### Reactive oxygen species measurement

Differentiated podocytes cultured with normal glucose (glucose 5.5 mM, supplemented with 24.5 mM mannitol as high osmolarity control) and high glucose (30 mM) were treated with 5% DMSO or puerarin for 24 hours. Cells were then incubated with 20 μm of DCFDA (Abcam, Cambridge, MA) for 30 min at 37 °C. Cells were viewed and photographed with a fluorescence microscopy with excitation and emission spectra of 495 nm and 529 nm respectively. DAPI was used for visualization of nuclear or phase-contrast pictures to visualize cells.

### NADPH oxidase activity measurement

The lucigenin-enhanced chemiluminescence method was used to monitor superoxide production as total Nox activity. Podocytes were washed twice with HBSS (CaCl_2_-2H_2_O 1.25 mM, KCl 5.4 mM, KH_2_PO_4_ 0.5 mM, MgCL_2_-6H_2_O 0.5 mM, MgSO_4_-7H_2_O 0.7 mM, NaCl 0.136 mM, NaHCO_3_ 4 mM, Na_2_HPO_4_ 0.4 mM, D-Glucose 5.5 mM, pH 7.2) and the culture plate was snap-frozen. Cells were scraped with ice-cold HBSS containing protease inhibitor cocktail. Cell suspensions were homogenized with 100 strkes in a Dounce homozinizer on ice. Protein content was quantified. 30ug of proteins were transferred to each well followed by adding 25uM Lucigenin (Santa Cruz, Lake Forest, CA) and 200 uM NADPH (Cayman Chemical, Ann Arbor, MI). After 10 minutes at 37 C for dark adaptation, the light emission was recorded. The NOX activity was expressed as relative light units per milligram of cellular protein in cells.

### Podocyte transfection

Podocytes were transfected with 5 μg of empty vector or corresponding vectors as indicated in the figures using Viafect reagent (Promega, E4981; Madison, WI) according to the manufacturer’s protocol.

### Western blot

Cells were lysed in M-PER mammalian protein extraction reagent (ThermoFisher, Waltham, MA) containing protease and phophastase inhibitor cocktail. Protein was separated on SDS-PAGE and transferred to PVDF membranes. Proteins were detected using specific antibodies: phospho-p65 (1:1000): Abcam, ab28856; total p65 (1:1000): Cell Signaling, 4764; Nox4 (1:2000): ab133303; GAPDH (1:2000): Cell Signaling 2118; beta-Actin (1:3000): Sigma A5136. The secondary antibodies were obtained from Jackson Immunoresearch Inc. and used at 1:10,000. Images of whole blots are included in the Supplementary Figure.

### Real time PCR

RNA transcript quantification was performed by 7500 Real-Time PCR System. Gene level was normalized to glyceraldehyde 3-phosphate dehydrogenase (GAPDH) and expressed as fold change. The primer sets used are as follows: SIRT1 (5′-CACTGTAACTGGGGGCAACT-3′ and 5′-CACTTCTTGTCAGCGTCGAA-3′), Nephrin (5′-CAGCCTCTTGACCATCGCTAA-3′ and 5′-TGGTGGCCGTGCATTTG-3′), Podocin (5′-TGGAAGCTGAGGCACAAAGA-3′ and 5′-CCCCTTCGGCAGCAATC-3′), and GAPDH (5′-GGCATTGCTCTCAATGACAA-3′ and 5′-TGTGAGGGAGATGCTCAGTG-3′).

### Statistical analysis

Data are expressed as mean ± SEM. The unpaired t-test was used to comparison between groups or ANOVA followed by Bonferroni correction was used when comparing between groups for treatment conditions using the GraphPad Prism software. *P-*value < 0.05 was considered statistically significant.

## Electronic supplementary material

Supplementary File
