# Publisher Correction: Puerarin attenuates diabetic kidney injury through the suppression of NOX4 expression in podocytes

**DOI:** 10.1038/s41598-017-17925-7

**Published:** 2017-12-22

**Authors:** Xueling Li, Weijing Cai, Kyung Lee, Bohan Liu, Yueyi Deng, Yiping Chen, Xianwen Zhang, John Cijiang He, Yifei Zhong

**Affiliations:** 1 Division of Nephrology, Longhua Hospital, Shanghai University of Traditional Chinese Medicine, Shanghai, China; 2 Department of Medicine, Division of Nephrology, Icahn School of Medicine at Mount Sinai, NY, USA; 3 Renal Section, James J Peters VAMC, Bronx, NY USA

Correction to: *Scientific Reports* https://doi.org/10.1038/s41598-017-14906-8, published online 03 November 2017

The original HTML version of this Article contained a typographical error in the publication date ‘03 November 2017’ which was incorrectly given as ‘06 November 2017’. This has now been corrected in the HTML version of the Article.

