# Publisher Correction: Puerarin attenuates diabetic kidney injury through the suppression of NOX4 expression in podocytes

**DOI:** 10.1038/s41598-018-22371-0

**Published:** 2018-03-06

**Authors:** Xueling Li, Weijing Cai, Kyung Lee, Bohan Liu, Yueyi Deng, Yiping Chen, Xianwen Zhang, John Cijiang He, Yifei Zhong

**Affiliations:** 1 Division of Nephrology, Longhua Hospital, Shanghai University of Traditional Chinese Medicine, Shanghai, China; 2 Department of Medicine, Division of Nephrology, Icahn School of Medicine at Mount Sinai, NY, USA; 3 Renal Section, James J Peters VAMC, Bronx, NY, USA

Correction to: *Scientific Reports* 10.1038/s41598-017-14906-8, published online 03 November 2017

An error occurred after publication resulting in the inadvertent omission of the Article and Volume numbers from the PDF version of this Article. This error has now been corrected in the PDF version of the Article; the HTML version was correct from the time of publication.

