# Supplementary material for: Puerarin attenuates diabetic kidney injury through the suppression of NOX4 expression in podocytes
Source: Sci Rep. 2017 Nov 3;7:14603. doi: 10.1038/s41598-017-14906-8 (PMC5668268; doi:10.1038/s41598-017-14906-8)

Supplementary Figures

Puerarin attenuates diabetic kidney injury through the suppression of NOX4 expression in podocytes

Xueling Li, Weijing Cai, Kyung Lee, Bohan Liu, Yueyi Deng, Yiping Chen, Xianwen Zhang, John Cijiang He, and Yifei Zhong

Supplementary Figure 1:: Full gel images for Figures 4-5

Figure 4C:

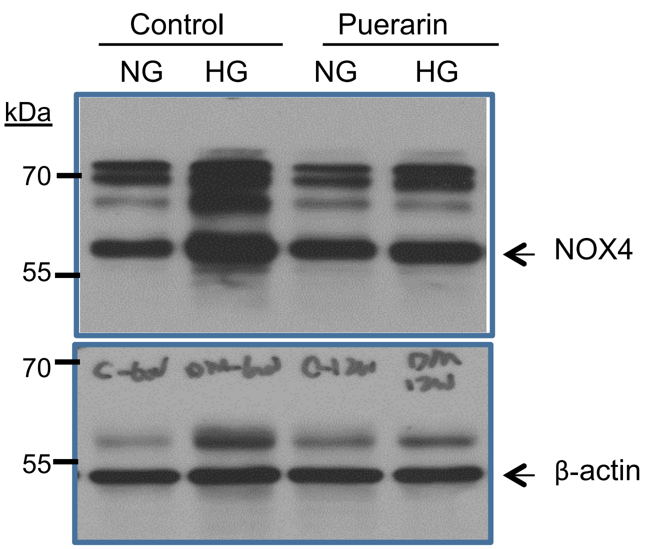

Figure 5A:

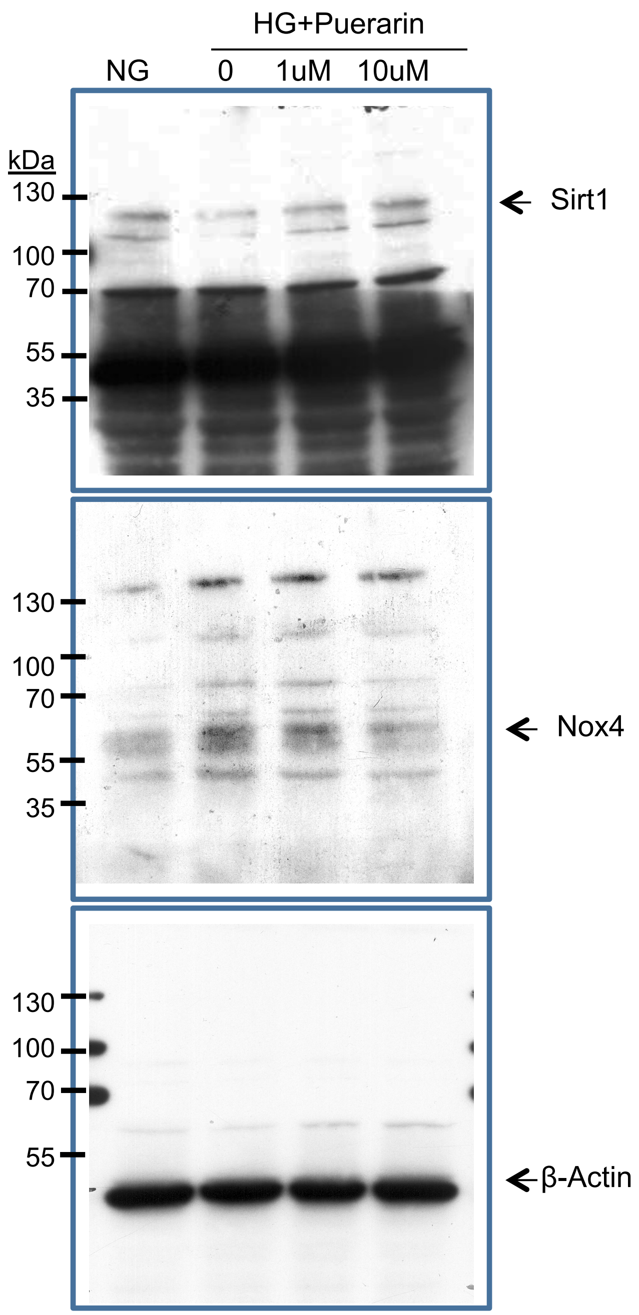

Supplementary Figure 1 (cont.)

Figure 5C:

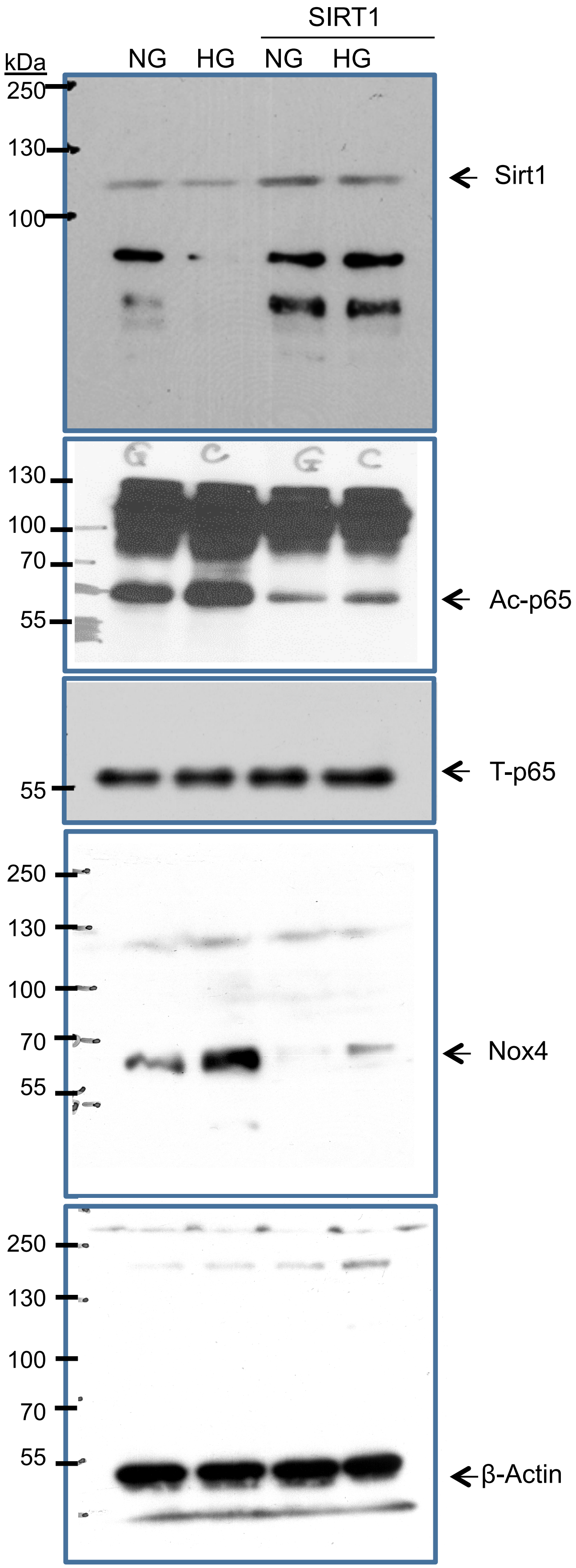

Figure 5E:

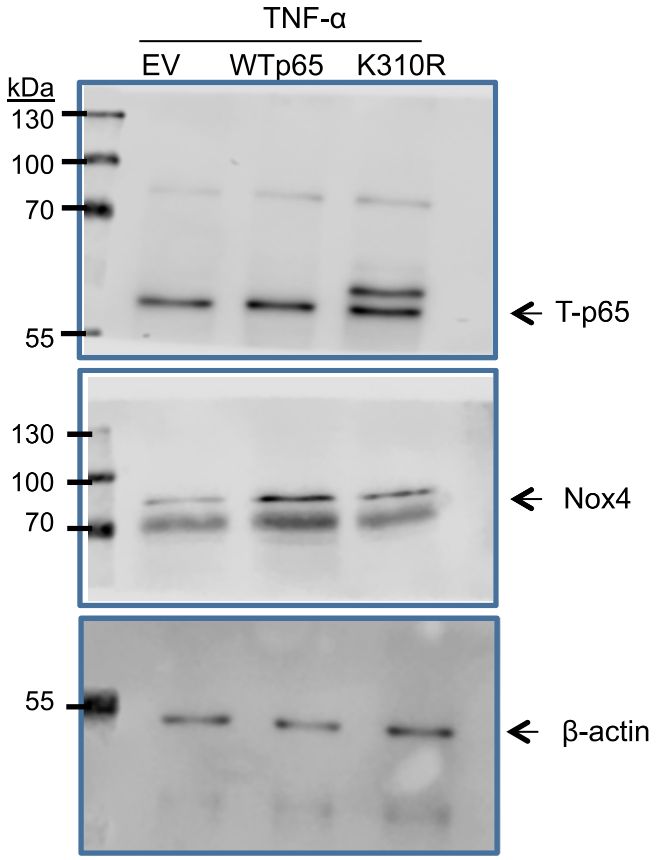

Supplement: Supplementary file 1 — Supplementary File [file 41598_2017_14906_MOESM1_ESM.pdf]
